# PE_PGRS38 Interaction With HAUSP Downregulates Antimycobacterial Host Defense *via* TRAF6

**DOI:** 10.3389/fimmu.2022.862628

**Published:** 2022-04-28

**Authors:** Jae-Sung Kim, Hyo Keun Kim, Euni Cho, Seok-Jun Mun, Sein Jang, Jichan Jang, Chul-Su Yang

**Affiliations:** ^1^ Department of Bionano Technology, Hanyang University, Seoul, South Korea; ^2^ Institute of Natural Science & Technology, Hanyang University, Ansan, South Korea; ^3^ Department of Molecular and Life Science, Hanyang University, Ansan, South Korea; ^4^ Center for Bionano Intelligence Education and Research, Hanyang University, Ansan, South Korea; ^5^ Molecular Mechanisms of Antibiotics, Division of Life Science, Research Institute of Life Science, Department of Bio & Medical Big Data (Brain Korea 21 Four Program), Gyeongsang National University, Jinju, South Korea

**Keywords:** *Mycobacterium tuberculosis* PE_PGRS38, *Mycobacterium smegmatis*, HAUSP, TRAF6, Macrophages, Ubiquitination

## Abstract

*Mycobacterium tuberculosis* (Mtb) is the causative pathogen of tuberculosis (TB), which manipulates the host immunity to ensure survival and colonization in the host. Mtb possess a unique family of proteins, named PE_PGRS, associated with Mtb pathogenesis. Thus, elucidation of the functions of PE_PGRS proteins is necessary to understand TB pathogenesis. Here, we investigated the role of PE_PGRS38 binding to herpesvirus-associated ubiquitin-specific protease (HAUSP, USP7) in regulating the activity of various substrate proteins by modulating their state of ubiquitination. We constructed the recombinant PE_PGRS38 expressed in *M. smegmatis* (Ms_PE_PGRS38) to investigate the role of PE_PGRS38. We found that Ms_PE_PGRS38 regulated the cytokine levels in murine bone marrow-derived macrophages by inhibiting the deubiquitination of tumor necrosis factor receptor-associated factor (TRAF) 6 by HAUSP. Furthermore, the PE domain in PE_PGRS38 was identified as essential for mediating TRAF6 deubiquitination. Ms_PE_PGRS38 increased the intracellular burden of bacteria by manipulating cytokine levels *in vitro* and *in vivo*. Overall, we revealed that the interplay between HAUSP and PE_PGRS38 regulated the inflammatory response to increase the survival of mycobacteria.

## Introduction

Tuberculosis (TB) is caused by *Mycobacterium tuberculosis* (Mtb). It is one of the well-known infectious diseases, resulting in a global morbidity and mortality burden. Mtb survive and persist in hosts by disturbing the host immune response through interactions with host proteins. The mechanisms of these interactions require elucidation to understand TB pathogenesis and develop new and effective therapeutic strategies (1–3).

Whole-genome analysis of Mtb strain H37Rv revealed that two specific protein families (PE and PPE) account for 10% of the genome. PE_PGRS is a subfamily of PE family proteins secreted by the ESX-5 secretion system and has 65 members that contain a PE and a PGRS domain. The PE domain is located in the N-terminal of PE_PGRS proteins, has a conserved PE (Pro-Glu) sequence, and is a similar length among the PE_PGRS proteins. The PGRS domain is situated in the C-terminal of PE_PGRS proteins and contains repetitive polymorphic GC-rich sequences (Gly-Gly-Ala and Gly-Gly-Asn) of variable lengths (4–6).

The characteristics of PE_PGRS protein are summarized as follows; First, PE_PGRS family proteins have intriguing traits that are most expressed in Mtb. Since many PE_PGRS proteins are distributed in the cell wall and cell membrane of Mtb, it is conceivable that they may interact with host proteins. Second, there is growing evidences that various PE_PGRS proteins were regulated the host immune signaling and cell deaths by mediating cytokine production, apoptosis factors, and autophagy in immune cells (7–11). Third, PE_PGRS proteins are related to cellular structure and the colony morphology. For example, Rv1818c-overexpressed *Mycobacterium smegmatis* cell size is elongated, but growth rate is normally (12). Fourth, some of PE_PGRS proteins are role as lipase to supply energy for survival and persistence of mycobacteria in macrophages (13, 14). Fifth, PE_PGRS proteins have high GC rich repeat tandem, and this feature could easily make recombination for source of antigenic variation. Furthermore, PE_PGRS proteins contain multiple calcium-binding GGXGXD/NXUX motifs, which could be regulate host calcium-dependent interactions (15–17). In summary, the few reported properties of PE_PGRS protein are related to the pathogenesis of Mtb. However, the functions of various other PE_PGRS proteins need to be elucidated.

HAUSP (USP7) is a deubiquitinase (DUB) that deubiquitinates and mediates the expression and function of several cellular and viral protein substrates. Infected cell polypeptide 0, a herpes simplex virus type 1 regulatory protein, interacts with HAUSP to regulate the viral lytic cycle (18). Furthermore, Epstein–Barr nuclear antigen 1 and viral interferon regulatory factors 1 and 4 bind to HAUSP in viral infections (19–21). HAUSP also interacts with various partners in cells to regulate the numerous proteins that mediate cellular processes, including chromosome segregation, cell cycle progression, gene expression, protein localization, DNA repair, kinase activation, protein degradation, inflammation, and apoptosis (22, 23).

Because the roles of many PE_PGRS proteins in host immune cells remain un elucidated, our study used mass spectrometry analysis and immunoprecipitation of 293T cells and bone marrow-derived macrophages (BMDMs) to reveal that the function of PE_PGRS38, a binding partner of HAUSP. Furthermore, we observed that the PE domain of PE_PGRS38 was essential for HAUSP interaction. We constructed recombinant *M. smegmatis* (Ms) that heterologously expressed PE_PGRS38 (Ms_PE_PGRS38) so that we could identify its function in the host. Ms_PE_PGRS38 increased the intracellular survival of bacteria and downregulated cytokine levels, including tumor necrosis factor-α (TNF-α), interleukin (IL)-6, IL-10, and IL-1β. The association of PE_PGRS38 with HAUSP inhibited deubiquitination of K48 polyubiquitin (K48-polyUb) and degradation of TRAF6. Furthermore, PE_PGRS38-expressing recombinant Ms enhanced the number of intracellular bacteria and reduced the cytokine levels in macrophages and mice. Thus, our findings demonstrated that PE_PGRS38 interaction with HAUSP in the host is associated with mycobacteria virulence.

## Results

### HAUSP Was Upregulated in Pulmonary TB Patients

**Figure 1:**
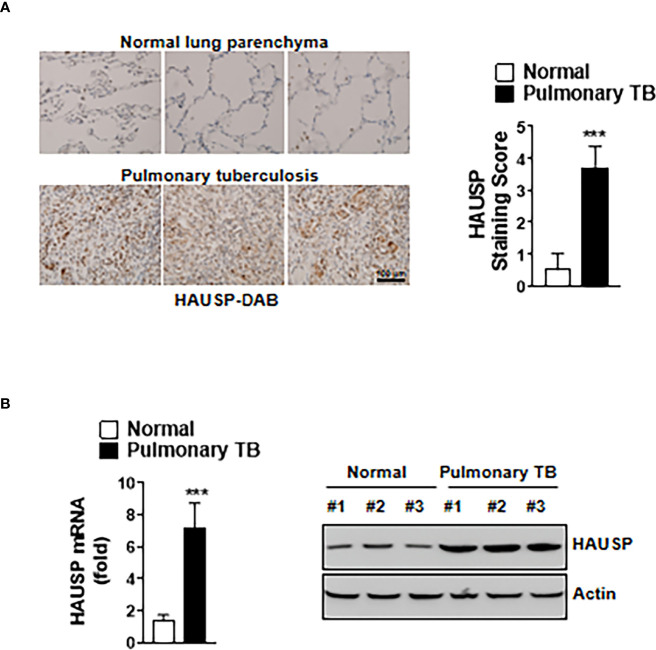
The expression of HAUSP was increased in pulmonary TB patients. **(A)** Immunohistochemistry images of HAUSP in the lungs of normal and pulmonary TB patients (Left) and staining score of HAUSP (Right). Scale Bar, 100μm **(B)** The expression level of mRNA and protein of HAUSP. The data are representative of four independent experiments with similar results **(A, B)**. Significant differences (***p < 0.001) are compared with the lungs of normal group (two-tailed Student’s t-test with Bonferroni adjustment).

We investigated HAUSP expression in TB using immunochemistry to stain for HAUSP in lung tissue sections from normal and TB patients. The HAUSP intensity score was approximately four times higher in lung tissue of TB patients compared with that of normal patients (**Figure 1A**). To examine the level of HAUSP in other disease, we additionally subjected immunochemistry in the lung of asthma and lung cancer patients. The level of HAUSP was increased in the lung of asthma and lung cancer patients compared to those of normal patients (**Figure S1**). Furthermore, the levels of HAUSP mRNA and protein were significantly higher in TB patients (**Figure 1B**). Collectively, these results indicated HAUSP upregulation in pulmonary TB.

### HAUSP Directly Interacted With Mtb PE_PGRS38

**Figure 2:**
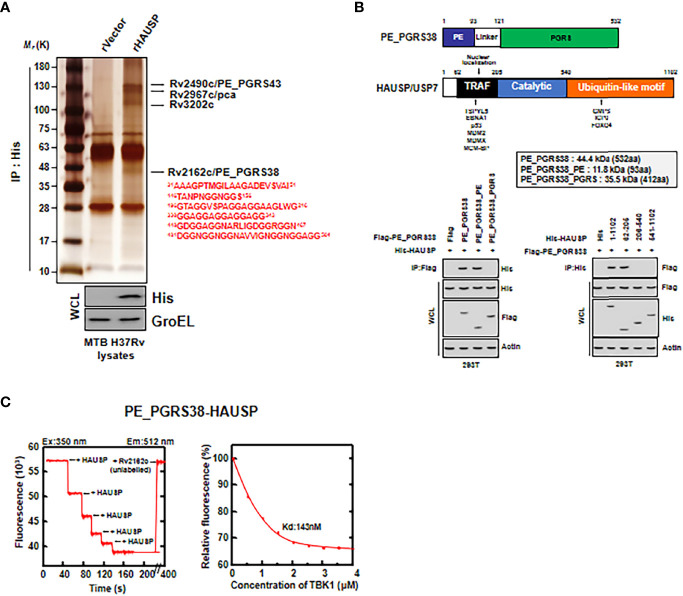
HAUSP directly bound to PE_PGRS38. **(A)** Identification of PE_PGRS38 as a binding partner of HAUSP. rVector or rHAUSP is treated in Mtb H37Rv lysates and incubated for 2 h and subjected to IP with αHis. Binding partners were confirmed by silver staining and mass spectrometry analysis. Whole cell lysate (WCL) was immunoblotted by αHis and αGroEL. **(B)** Binding mapping. Schematic diagrams of the structures of PE_PGRS38 and HAUSP (upper). Flag, Flag-PE_PGRS38 or its truncated mutant constructs were transfected with His, His-HAUSP or its truncated mutant constructs in 293T cells. After 48h transfection, 293T cells were used for IP with αFlag, followed by IB with αHis. WCLs were used for IB with αHis, αFlag, and αActin. **(C)** Titration of fluorescently labelled HAUSP with PE_PGRS38 (left), with K_d_ (143 nM), determined by curve fit analysis (right). The data are representative of four independent experiments with similar results **(A–C)**.

To investigate which Mtb antigens were HAUSP-binding partners, we subjected recombinant HAUSP (rHAUSP) and Mtb H37Rv strain lysate to coimmunoprecipitation. Then, the purified rHAUSP complexes were identified by mass spectrometry analysis. Several Mtb proteins were detected as binding partners of rHAUSP, including Rv2490c (PE_PGRS43), Rv2967c (pyruvate carboxylase, pca), Rv3202c (DNA helicase), and Rv2162c (PE_PGRS38) (**Figure 2A**). This study focused on PE_PGRS38, a member of the PE_PGRS protein family solely expressed in mycobacteria (4, 6).

PE_PGRS38 is composed of a PE domain and a PGRS domain. We determined which PE_PGRS38 domain was essential for interacting with HAUSP in 293T cells. First, we transfected Flag-tagged PE_PGRS38 and His-tagged HAUSP or their truncated mutants into 293T cells, followed by immunoprecipitation (IP). The PE domain of PE_PGRS38 and the TRAF domain of HAUSP were identified as important for PE_PGRS38-HAUSP interaction (**Figure 2B**). Additionally, the results of a fluorescence binding assay with fluorescently labeled rHAUSP and unlabeled recombinant PE_PGRS38 were used as a measure of the *in vitro* interaction between HAUSP and PE_PGRS38 and showed an intimately high affinity (HAUSP, 350 nm; Rv2162c, 512 nm; K_d_ = 143 nM) (**Figure 2C**). Thus, PE_PGRS38 directly binds to HAUSP, and the PE domain of PE_PGRS38 is essential for PE_PGRS38-HAUSP interaction.

### PE_PGRS38 Upregulates Bacterial Survival by Regulating Cytokine Production Through HAUSP Interaction

**Figure 3:**
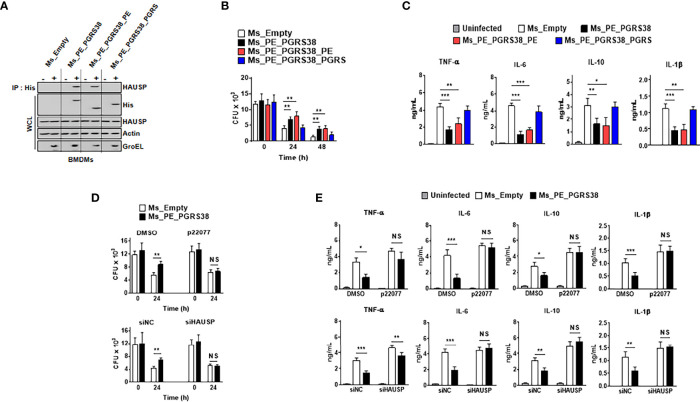
PE_PGRS38 enhanced the survival of intracellular bacteria with attenuating the production of cytokines in BMDMs. BMDMs were infected with Ms_Empty, Ms_PE_PGRS38, Ms_PE_PGRS38_PE, and Ms_PE_PGRS38_PGRS at an MOI of 5. **(A)** BMDMs were lysed after 24 h post-infection, followed by IP with αHis. Immunoblotting was subjected with αHAUSP. WCLs were used for αHis, αHAUSP, αActin, and αGroEL. **(B)** Survival of intracellular bacteria burden was estimated by CFU plating in indicated times. **(C)** After 24 h post-infection, supernatants of BMDMs were used for analysis of the level of TNF-α, IL-6, IL-10, and IL-1β. **(D, E)** BMDMs were pretreated with p22077 (5 μM) for 2 h or siHAUSP (20 μM) for 2 days. After treatment of p22077 or siHAUSP, BMDMs were infected with Ms_Empty or Ms_PE_PGRS38 for 3 h. **(D)** Intracellular bacteria burden were calculated and **(E)** supernatants of BMDMs were used for analysis of the level of TNF-α, IL-6, IL-10, and IL-1β by ELISA. The data are representative of four independent experiments with similar results **(A–E)**. Significant differences (**p < 0.01; ***p < 0.001; NS, No Significant) are compared with the Ms_Empty infected BMDMs (two-tailed Student’s t-test with Bonferroni adjustment).

PE_PGRS proteins mediate the host immune response and enhance the intracellular residue of bacteria (24–30). We infected BMDMs with Ms_PE_PGRS38 or its truncated domain to determine the role of PE_PGRS38 in macrophages. First, we constructed plasmids expressing PE_PGRS38 or its truncated mutant to generate Ms_PE_PGRS38 (**Figure S2A**). PE_PGRS38 and its truncated domains were amplified from the Mtb H37Rv genome using specific primers (**Figure S2B**). Strains Ms_PE_PGRS38, Ms_PE_PGRS38_PE, and Ms_PE_PGRS38_PGRS expressed mRNA and protein of PE_PGRS38 or its truncated domains following transformation into a pMV262 vector (**Figures S2C, D**). Expression of PE_PGRS38 or PE_PGRS38_PGRS domain decreased rate of replication in bacteria compared to Ms_Empty (**Figure S2E**). The morphology of colony was slightly different, but the sizes of colony are similar among Ms (**Figure S2F**). Then, we infected BMDMs with recombinant Ms to investigate the endogenous interaction of PE_PGRS38-HAUSP. Ms_PE_PGRS38 and Ms_PE_PGRS38_PE associated with HAUSP, but Ms_PE_PGRS38_PGRS did not (**Figure 3A**). Because Ms is a nonpathogenic mycobacterium, intracellular Ms is trapped in phagosome and unable to escape from phagosome. We further examined that PE_PGRS38 and HAUSP interacted in phagosome. We fractionated phagosome from BMDMs and immunoprecipitated in phagosomal fraction. Intriguingly, PE_PGRS38 bound to HAUSP in phagosome in BMDMs (**Figure S3A**). PE_PGRS38 and the PE domains of PE_PGRS38 increased the survival of bacteria in macrophages and decreased the production of cytokines, such as TNF-α, IL-6, IL-10, and IL-1β, but this was not observed with the PGRS domain of PE_PGR38 (**Figures 3B, C**). Additionally, to determine whether the role of PE_PGRS38 was linked to HAUSP, we limited HAUSP using small interfering (si)RNA or the HAUSP inhibitor P22077 in BMDMs. We validated that siHAUSP significantly reduced HAUSP expression in BMDMs (**Figure S3B**). HAUSP knockdown or inhibition did not cause any significant differences in the number of intracellular bacteria and the cytokine levels in BMDMs between Ms_Empty and Ms_PE_PGRS38 (**Figures 3D, E**). Collectively, these results showed that PE_PGRS38 enhanced the survival of bacteria by downregulating cytokine production in macrophages in an HAUSP-dependent manner.

### PE_PGRS38 Inhibits K48-polyUb Deubiquitination in TRAF6 *via* HAUSP Interaction

**Figure 4:**
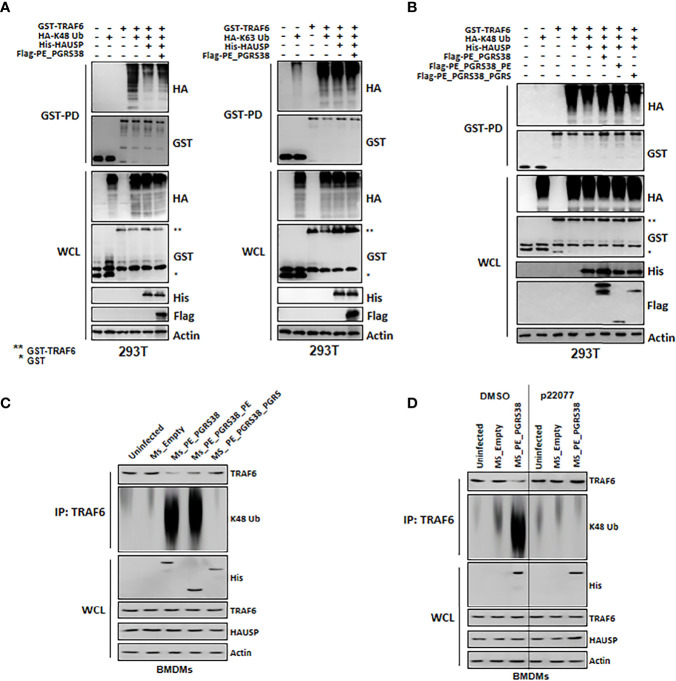
PE_PGRS38 declined deubiquitination of K48-polyUb in TRAF6. **(A)** 293T cells were transfected with GST or GST-TRAF6, HA, HA-K48 or K63 Ub, His or His-HAUSP, and Flag or Flag-PE_PGRS. After 48h transfection, 293T cells were used for GST-pulldown and immunoblotting with αHA and αGST. WCLs were used for IB with αHA, αGST, αHis, αFlag, and αActin. **(B)** 293T cells were transfected with GST or GST-TRAF6, HA or HA-K48, His or His-HAUSP, and Flag, Flag-PE_PGRS, Flag-PE_PGRS38_PE, or Flag-PE_PGRS38_PGRS. After 48h transfection, 293T cells were used for GST-pulldown and immunoblotting with αHA and αGST. WCLs were used for IB with αHA, αGST, αHis, αFlag, and αActin. **(C)** BMDMs were infected with Ms_Empty, Ms_PE_PGRS38, Ms_PE_PGRS38_PE, and Ms_PE_PGRS38_PGRS for 3 h. After 24h post-infection, BMDMs were lysed and immunoprecipitated by αTRAF6, followed by αTRAF6 and αK48 Ub. WCLs were used for IB with αHis, αTRAF6, αHAUSP, and αActin. **(D)** BMDMs were pretreated with p22077 (5 μM) for 2 h or siHAUSP (20 μM) for 2 days. After treatment of p22077 or siHAUSP, BMDMs were infected with Ms_Empty or Ms_PE_PGRS38 for 3 h. After 24h post-infection, BMDMs were lysed and immunoprecipitated by αTRAF6, followed by αTRAF6 and αK48 Ub. WCLs were used for IB with αHis, αTRAF6, αHAUSP, and αActin. The data are representative of four independent experiments with similar results **(A–D)**. *Glutathioine S-transferase (GST); **Glutathionine S-transferase (GST) tagged TRAF6.

We demonstrated that cytokine levels decreased in BMDMs infected with Ms_PE_PGRS38 or Ms_PE_PGRS38_PE (**Figure 3**). This suggested that PE_PGRS38 might regulate cytosolic upstream factor, which is an HAUSP substrate, in NF-κB signaling pathways. HAUSP regulates the level or function of proteins *via* deubiquitination (22, 23) and was also reported to deubiquitinate K48-polyUb in TRAF6 and mediate cytokine production (31). Thus, we investigated whether PE_PGRS38 regulated the role of HAUSP in the deubiquitination of K48-polyUb in TRAF6. We transfected 293T cells with GST-TRAF6, HA-K48 or K63 Ub, His-HAUSP, and Flag-PE_PGRS38 and then subjected them to a GST-pulldown assay. K48-polyUb in TRAF6 was deubiquitinated by HAUSP, and PE_PGRS38 inhibited the deubiquitination of K48-polyUb in TRAF6 but not of K63-polyUb in TRAF6 (**Figure 4A**). We performed further investigations using the truncated domains of PE_PGRS38, and deubiquitination of K48-polyUb in TRAF6 was repressed in the full-length PE_PGRS38 and the PE domain of PE_PGRS38 (**Figure 4B**). In BMDMs, deubiquitination of endogenous K48-polyUb in TRAF6 was also declined by PE_PGRS38 and the PE domains of PE_PGRS38 (**Figure 4C**). However, the deubiquitination of K48-polyUb in TRAF6 was not modulated by PE_PGRS38 or PE_PGRS38_PE following treatment with P22077 or HAUSP knockdown in BMDMs (**Figure 4D**). Overall, PE_PGRS38 suppressed K48-polyUb deubiquitination in TRAF6 in an HAUSP-dependent manner.

### PE_PGRS38 Increases the Intracellular Bacterial Burden and Downregulates Cytokine Levels in Mice

**Figure 5:**
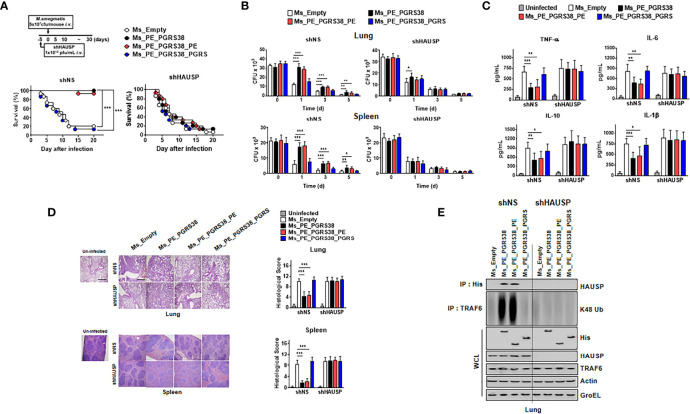
PE_PGRS38 elevated the bacterial burden and reduced the level of cytokines in mice. **(A)** Schematic of the infection of Ms_Empty, Ms_PE_PGRS38, Ms_PE_PGRS38_PE, and Ms_PE_PGRS38_PGRS (5x10^7^ cfu/mouse) in shNS or shHAUSP mice (upper). The survival of mice was monitored for 20 days; mortality was measured for n = 10 mice per group (lower). Statistical differences were compared with the Ms_Empty infected mice are indicated (Log-rank test). The data are representative of two independent experiments with similar results. **(B–E)** Mice were intravenously infected with Ms_Empty, Ms_PE_PGRS38, Ms_PE_PGRS38_PE, Ms_PE_PGRS38_PGRS (1x10^6^ cfu/mouse) in shNS or shHAUSP mice. **(B)** The bacterial burden in the lungs and spleens in shNS or shHAUSP mice for indicated times. **(C)** The level of TNF-α, IL-6, IL-10, and IL-1β in 3 days post-infected mice serum was measured by ELISA. **(D)** Representative H&E staining of the lung and spleen (left) (n = 10 mice per group). Histopathology scores were obtained from H&E staining images as described in methods (right) were determined at 3 days post-infection in shNS or shHAUSP mice. Scale bar, 500 μm. **(E)** Lung of 3 days post-infected mice were lysed and immunoprecipitated by αHis or αTRAF6, followed by IB with αHAUSP and αK48 Ub. WCLs were used for IB with αHis, αHAUSP, αTRAF6, αActin, and αGroEL. The data are representative of two independent experiments with similar results **(B–E)**. Significant differences (*p < 0.05; **p < 0.01; ***p < 0.001) are compared with the Ms_Empty infected mice (two-tailed Student’s t-test with Bonferroni adjustment).

We infected mice with Ms_Empty, Ms_PE_PGRS38, Ms_PE_PGRS38_PE, and Ms_PE_PGRS38_PGRS in short hairpin (sh)NS and shHAUSP *via* intravenous injection to investigate the role of PE_PGRS38 *in vivo*. Before infection, we validated the attenuation of HAUSP by lenti-shHAUSP, and HAUSP was significantly reduced in lungs and blood (**Figures S3A, B**). We used a high dose (5 x 10^7^ cfu/mouse) of Ms to infect the mice so that we could observe their survival. Ms_Empty and Ms_PE_PGRS38_PGRS significantly decreased the rate of survival, but this was not observed with Ms_PE_PGRS38 and Ms_PE_PGRS38_PE. We assumed that the infection of a high dose of Ms would induce hyperinflammation in mice, but PE_PGRS38 inhibited excessive cytokine secretion and increased the survival rate. In contrast, shHAUSP mice showed no significant difference compared with Ms_Empty mice in all groups (**Figure 5A**).

We measured the bacterial burden in lung and spleen tissue at 0, 1, 3, and 5 days post infection. Ms_PE_PGRS38 and Ms_PE_PGRS38_PE survived longer than Ms_Empty and Ms_PE_PGRS38_PGRS in lung and spleen tissue but not in shHAUSP mice (**Figure 5B**). Additionally, cytokine production and pathology scores were decreased in Ms_Empty- and Ms_PE_PGRS38_PE-infected shNS mice but not in shHAUSP mice (**Figures 5C, D**). K48-polyUb deubiquitination was decreased in Ms_PE_PGRS38- and Ms_PE_PGRS38_PE-infected shNS mice but not shHAUSP mice (**Figure 5E**). In summary, PE_PGRS38 enhanced the burden of intracellular bacteria and downregulated the level of cytokines through interaction with HAUSP. Intriguingly, PE_PGRS38 increased the survival of mice and decreased the pathogenesis in excessive inflammation caused by infection with a high dose of bacteria.

## Discussion

We found that the interaction of PE_PGRS38 with HAUSP affected the inflammatory response. The major findings of this study are as follows: (1) HAUSP is increased in TB patients; (2) PE_PGRS38 is directly associated with HAUSP in macrophages, and the PE domain in PE_PGRS38 is vital for HAUSP and PE_PGRS38 interaction; (3) PE_PGRS38 expressed in Ms decreases the cytokine levels and increases the burden of intracellular bacteria in BMDMs; (4) PE_PGRS38 inhibits K48-polyUb ubiquitination *via* association with HAUSP; and (5) PE_PGRS38 increases the bacterial burden and downregulates cytokine levels in mice.

HAUSP is a well-known fundamental DUB in the tumor suppressor p53-dependent pathway in viral infections. HAUSP interacts with p53 and p53 regulator MDM2 and regulates the degradation of both proteins under certain cellular conditions (32–34). Stabilization of p53 is linked to the activation of viral immunity, and the viral proteins of some viruses interact with HAUSP, which disturbs the p53 pathway and leads to successful viral infection (20, 35–37). However, it is unclear which Mtb proteins interact with HAUSP during mycobacterial infection. We found that HAUSP expression was increased in pulmonary TB patients, and HAUSP interacted with PE_PGRS38 proteins. These results suggested that HAUSP interaction with PE_PGRS38 plays a vital role in mycobacterial infection.

PE_PGRS38, encoded by *Rv2162c*, is widespread in a variety of pathogenic mycobacteria, such as *M. avium*, *M. gilvum*, and *M. leprae*, and in nonpathogenic mycobacteria (4). Additionally, PE_PGRS38 was regarded as a new target of capreomycin, which is a popular second-line treatment for TB and multidrug-resistant TB (38). However, the function of PE_PGRS38 during infection has not been previously reported. Most of the PE_PGRS proteins that have been previously studied were found to play a role in the regulation of the host immune system in mycobacterial infection. PE_PGRS29, PE_PGRS30, and PE_PGRS33 enhanced intracellular bacterial survival and modulated the secretion of proinflammatory cytokines, such as TNF-α, IL-6, and IL-12, in macrophages and mice (24, 26, 39, 40). PE_PGRS41 and PE_PGRS47 also protected Mtb by inhibiting various immune-related processes, including autophagy, antigen presentation, and phagosomal maturation (10, 28). In accordance with other PE_PGRS proteins, we identified novel functions of PE_PGRS38 that modulated the cytokine secretion and increased the bacterial burden in macrophages and mice by using heterologous expression of PE_PGRS38 in recombinant Ms.

TRAF6 is an intermediate protein in inflammatory-related signaling pathways, such as NF-κB, MAPK, Wnt/β-catenin, and c-myc. Poly-K63 ubiquitination in TRAF6 recruits downstream proteins, including TAB2, TAK1, NEMO, IKKα/β, and TRAF2/3. Subsequently, TRAF6 forms a complex with the adaptor proteins and activates the transcriptional factors to express genes involved in multiple cellular processes. Because TRAF6 is a fundamental bridge in various signaling pathways, the regulation of its cytosolic level is crucial to maintaining cellular homeostasis (41–43). Some studies reported that several mycobacteria antigens interacted with TRAF6 and regulated the activation of inflammation in the host. Rv0222, which is a serodiagnostic protein for tuberculosis, was associated with anaphase promoting complex subunit 2 (ANAPC2) and promoted K11-linked-ubiquitination in K76 of Rv0222. This ubiquitination in Rv0222 reduced TRAF6 activation through blocking the K63-linked ubiquitination in TRAF6 and downregulated the level of inflammatory cytokines with increase the survival of Mtb. Deletion of K76 in Rv0222 or knockdown of ANAPC2 impaired the function of Rv0222 and attenuated the virulence of Mtb (44). Furthermore, Rv2626c, a promising vaccine candidate of tuberculosis, directly bound to TRAF6 and inhibited K63-linked polyubiquitination in TRAF6. C-terminal 123-131 amino acids of Rv2626c is essential for interaction with TRAF6 and enhanced macrophage recruitment, phagocytosis, polarization of M2 macrophages and elimination of bacteria (45). HAUSP is a positive regulator of TRAF6, with deubiquitinating K48-polyUb for inhibition of TRAF6 degradation. In macrophages, HAUSP increased TRAF6 stability after lipopolysaccharide treatment and enhanced inflammatory cytokine production. Treatment with HAUSP inhibitor P22077 suppressed TRAF6 deubiquitination and the level of cytokines (31). Another study reported that *Helicobacter pylori* infection, which causes gastric disease and cancer, regulated HAUSP expression and was dependent on a pathogenicity island (PAI) related to chronic inflammation in gastric epithelial cells. PAI-mutant *H. pylori-*infected gastric epithelial cells showed decreased levels of HAUSP and IL-8 production compared with wild-type *H. pylori*-infected cells (46). In our study, we found that the interaction between HAUSP and PE_PGRS38 upregulated the degradation of TRAF6 by blocking deubiquitination of K48-polyUb in TRAF6. TRAF6 regulation induced alterations in cytokine levels and increased bacterial persistence in macrophages and mice. Thus, we suggest that PE_PGRS38 may be a potential pathogenic Mtb antigen for the regulation of cytokine production by directly binding to HAUSP.

In summary, we found that PE_PGRS38 might be related to Mtb virulence by prolonging persistence and colonization and regulating cytokine production through HAUSP interaction and enhancing the degradation of TRAF6 by blocking the deubiquitination of K48-polyUb in TRAF6. These results suggest the relevance of PE_PGRS38 in TB pathogenesis. Further investigations of PE_PGRS proteins are necessary to understand their role in TB.

## Materials and Methods

### Mice and Cell Culture

Wild-type C57BL/6 mice were purchased from Samtako Bio Korea (Gyeonggi-do, Korea). Primary bone marrow–derived macrophages (BMDMs) were isolated from C57BL/6 mice and cultured in DMEM for 3–5 d in the presence of M-CSF (416-ML, R&D Systems; NE Minneapolis, MN, USA), as described previously (47). HEK293T (ATCC-11268, American Type Culture Collection; Manassas, VA, USA) cells were maintained in DMEM or RPMI1640 (Gibco, NY, USA) containing 10% FBS (Gibco, NY, USA), sodium pyruvate, nonessential amino acids, penicillin G (100 IU/ml), and streptomycin (100 μg/ml). All animal-related procedures were reviewed and approved by the Institutional Animal Care and Use Committee of the Hanyang University (protocol 2020-0060).

### Construction of Recombinant *M. smegmatis* Strains

The intact PE_PGRS38 and its deletion genes were amplified from Mtb H37Rv genome using gene-specific primers (forward with BamH I site–5′ - reverse with EcoR I site-5′ -). The amplified PE_PGRS38 gene was cloned into downstream of the hsp60 promoter of pMV262 vector. The recombinant plasmids were transformed into *M. smegmatis* mc^2^155 by electroporation. The transformed recombinant *M. smegmatis* strains were selected on Middlebrook (MB) 7H10 agar (Difco Laboratories; Franklin lakes, NJ, USA) containing 10% OADC (Difco Laboratories; Franklin lakes, NJ, USA) as growth supplement, 0.05% (v/v) Tween 80, 0.5% (v/v) glycerol, and 50 μg/ml kanamycin. Selected colonies were cultured in MB 7H9 broth supplement with 10% OADC, 0.05% (v/v) Tween 80, 0.5% (v/v) glycerol, and 50 μg/ml kanamycin. The expression of PE_PGRS38 in constructs were confirmed by qPCR, and the positive recombinant strains were stored with sterile 20% glycerol at − 80°C for further use. All recombinant *M. smegmatis*-related procedures were reviewed and approved by Institutional Biosafety Committee of the Hanyang University (HY-IBC-2020-01).

### *In Vitro* and *In Vivo* Infection With Recombinant *M. smegmatis*

For *in vitro* experiments, BMDMs were infected with Ms_Empty, Ms_PE_PGRS38, Ms_PE_PGRS38_PE or Ms_PE_PGRS38_PGRS at an MOI of 5 for 3 h. Then, cells were washed with PB+inS to remove extracellular bacteria, supplied with fresh medium with 50 ug/ml gentamicin (Manilbio; Jeollabuk-do, Korea), and incubated at 37°C for indicated time points. In time point, the infected macrophages were washed 2 times with PBS and lysed in 1 ml NP-40. The cell lysates were diluted, and each dilution were plated on 7H10 agar plates and incubated at 37°C. After 3 days, colonies on plates were counted and the survival rate was compared to Ms_Empty.

For *in vivo* experiments, female SPF C57BL/6 mice were 6-8-weeks old during the experiments and were age- and sex-matched in each experiment. No additional randomization or blinding was used to allocate experimental groups. Before 5 days for Ms infection, mice were infected with lenti-shNS and lenti-shHAUSP *via* intravenous injection (*i.v*). After 5 days, mice were *i.v.* injected with Ms_Empty, Ms_PE_PGRS38, Ms_PE_PGRS38_PE or Ms_PE_PGRS38_PGRS (5×10^7^ cfu/mouse) and survival rate was measured for 20 days. To count the intracellular bacteria in the lungs and spleens, mice were sacrificed on indicated times and homogenized the lungs and spleens with 300 μl of 0.5% NP-40 in PBS. Homogenates were centrifuged on 500 x g for 2 mins and plated on 7H10 agar plates and incubated at 37°C. After 3 days, colonies on plates were counted and the survival rate was compared to Ms_Empty. All animal-related procedures were reviewed and approved by the Institutional Animal Care and Use Committee of the Hanyang University (protocol 2019-0081).

### Recombinant Protein

To obtain Mtb H37Rv strain-derived recombinant PE_PGRS38 (rPE_PGRS38) and HAUSP (rHAUSP) protein, PE_PGRS38 and HAUSP sequence were cloned with an N-terminal 6xHis tag into the pET22b Vector (Novagen; Madison, WI, USA) and induced, harvested, and purified from *Escherichia coli* expression strain BL21(DE3)pLysS as described previously (47), in accordance with the standard protocols recommended by Novagen. rPE_PGRS38 and rHAUSP was dialyzed with permeable cellulose membrane and tested for lipopolysaccharide contamination with a *Limulus* amebocyte lysate assay (BioWhittaker; Walkersville, MD, USA) and contained < 20 pg/ml at the concentrations of rPE_PGRS38 and rHAUSP proteins used in the experiments described here.

### Mass Spectrometry

To identify HAUSP-binding proteins, Mtb H37Rv lysates were incubated with rVector or rHAUSP for 2 h. Lysates were precleared with protein A/G beads at 4°C for 2 h. Precleared lysates were mixed with αHis antibody-conjugated with agarose beads for 4 h at 4°C. Precipitates were washed extensively with washing buffer. Proteins bound to beads were eluted and separated on a Nupage 4-12% Bis-Tris gradient gel (Invitrogen; Waltham, MA, USA). After silver staining (Invitrogen; Waltham, MA, USA), specific protein bands were excised and analyzed by ion-trap mass spectrometry at the Korea Basic Science Institute (Seoul, Korea) Mass Spectrometry facility, and amino acid sequences were determined by tandem mass spectrometry and database searches.

### Antibodies and Reagents

Abs specific for Actin (I-19) were purchased from Santa Cruz Biotechnology (Dallas, Texas, USA). The antibodies to TRAF6 (ab137452) and GroEL (ab90522) were purchased from Abcam (Cambridge, UK). The antibody to GST (91G1), HAUSP (D17C6) and K48 Ub (D9D5) were purchased from Cell Signaling Technology (Danvers, MA, USA) and Flag (M185-3L), His (D291-3), and HA (M180-3) were purchased from MBL life science (Sunnyvale, CA, USA). P22077 (S7133) were purchased from Sellekchem (Houston, TX, USA). siHAUSP (sc-77373) were purchased from Santa cruz Biotechnology. RNAiMAX (13778) were purchased from Invitrogen (Waltham, MA, USA). shHAUSP were purchased from Horizon discovery (Waterbeach, UK).

### Interaction Kinetic Analyses of the HAUSP and PE_PGRS38

The interaction of HAUSP-PE_PGRS38 were monitored using a Fluoromax-4 spectrofluorometer (HORIBA Scientific, Piscataway, NJ, USA), and was performed as previously described (48). Briefly, HAUSP was labelled with BODIPY FL Iodoacetamide (ThermoFisher Scientific; Waltham, MA, USA), according to the manufacturer’s instructions. Labelled HAUSP was excited at 350 nm, and detection was through a cutoff filter at 512 nm. Fluorescently labelled HAUSP was titrated with unlabeled PE_PGRS38 for the kinetic analysis. The excitation and emission wavelengths used were 498 mm and 518 nm, respectively. The data obtained were fitted using the program Grafit. All fluorescence measurements were performed at 25°C in 30 mM Tris, pH 7.4, 150 mM NaCl and 1 mM dithiothreitol.

### Enzyme-Linked Immunosorbent Assay

Cell culture supernatants and mice sera were analyzed for cytokine content using the BD OptEIA ELISA set (BD Pharmingen; San diego, CA, USA) for the detection of TNF-α, IL-6, IL-10, and IL-1β. The plates were coated with capture antibody for O.N at 4 ˚C. After coating, samples were incubated for 2 h at RT, followed by treating the detection antibody in plates for 1 h at RT. After treating detection antibody, streptavidin-HRP was incubated in plates for 30 mins at RT, and TMB substrate solution is added to each well for 30 mins at RT. Next, stop solution is added to each well and the plates were measured at 450 nm using MMR SPARK^®^ Microplate Reader (Männedorf, CH). All assays were performed as recommended by the manufacturer.

### Plasmid Transfection

293T cells were seeded into 6 wells dish and incubated overnight. 293T cells were transfected with plasmid using lipofectamine 2000 (Aptabio; Gyeonggi-do, Korea) according to the manufacturer’s instructions. Culture medium was changed 4 h after transfection. After 24 h, cells were lysed by NP-40 buffer and collected for subsequent experiments.

### Glutathione S-Transferase (GST) Pulldown, Immunoblot, and Immunoprecipitation Analysis

GST pulldown, immunoprecipitation, and immunoblot assays were performed as described previously (45, 49). For GST pulldown, 293T cells were harvested and lysed in NP-40 buffer supplemented with a complete protease inhibitor cocktail (Roche; Basal, CH). After centrifugation, the supernatants were precleared with protein A/G beads at 4°C for 2 h. Pre-cleared lysates were mixed with a 50% slurry of glutathione-conjugated Sepharose beads (Amersham Biosciences; Amersham, UK), and the binding reaction was incubated for 4 h at 4°C. Precipitates were washed extensively with lysis buffer. Proteins bound to glutathione beads were eluted with sodium dodecyl sulfate (SDS) loading buffer by boiling for 5 min.

For immunoprecipitation, 293T cells, BMDMs, and mice lung were lysed in NP-40 buffer supplemented with a complete protease inhibitor cocktail (Roche; Basal, CH). After pre-clearing with protein A/G agarose beads for 1 h at 4°C, whole-cell lysates were used for immunoprecipitation with the indicated antibodies. Generally, 1-4 μg of commercial antibody was added to 1 ml of cell lysates and incubated at 4°C for 8 to 12 h. After the addition of proteins A/G agarose beads for 6 h, immunoprecipitates were extensively washed with lysis buffer and eluted with SDS loading buffer by boiling for 5 min.

For immunoblotting (IB), polypeptides were resolved by SDS-polyacrylamide gel electrophoresis and transferred to a PVDF membrane (Bio-Rad, Hercules, CA, USA). Immuno detection was achieved with specific antibodies. Antibody binding was visualized by chemiluminescence (ECL; Millipore, Burlington, MA) and detected by a Vilber chemiluminescence analyzer (Fusion SL 3; Vilber Lourmat).

### Quantitative Real-Time Polymerase Chain Reaction (PCR)

Total RNA was extracted from cells using a RNeasy RNA extraction Mini-Kit (Qiagen; Venlo, Netherlands). cDNA was synthesized using an Enzynomics kit (Enzynomics; Daejeon, Korea) and quantitative PCR was performed using SYBR Green PCR Master Mix (Enzynomics; Daejeon, Korea). Real-time PCR was performed using a QuantStudio™ 3 (ABI, Waltham, MA, USA), according to the manufacturer’s instructions. Data were normalized to the expression of qcrB or β-actin. Relative expression was calculated using the delta–delta Ct method. The sequences of the primers were as follows: PE_PGRS38 (Forward: gtgcagcagaccctgtca; Reverse: gtcggcgccgttgccgat), rpoB (Forward: tctccgagatcatgatgggc; Reverse: tcgtagcgcttctccttgaa), mHAUSP (Forward: tcaagtctcaaggttataggga; Reverse: gctgatagtaaagtttcttagg), mβ-Actin (Forward: agatcaagatcattgctcctc; Reverse: gtgtaaaacgcagctcagta).

### Study Population of Human Normal and TB Patients

Two types of samples were included in this study. All participants, as both patients and human normal, provided informed consent, and the patients were enrolled based on their diagnosis prior to chemotherapy treatment. We collected lung biopsy from 20 individuals: 10 human lung (median age 43.8 ± 15.2 y; male 45.1%) and 10 TB patients (median age 51.6 ± 13.8 y; male 48.2%). All participants provided written informed consent regarding the use of their clinical data for research purposes.

### Histology and Immunohistochemistry

For H&E staining of tissue sections, mouse spleens and lungs were fixed in 10% formalin and embedded in paraffin. Paraffin sections (4 μm) were cut and stained with hematoxylin and eosin. Histopathologic score was established based on the numbers and distribution of inflammatory cells and the severity of inflammation within the tissues (50, 51) in which a board-certified pathologist (Dr. Min-Kyung Kim, Kim Min-Kyung Pathology Clinic, Seoul, Korea) independently scored each organ section without prior knowledge of the treatment groups. A histological score ranging from 0-4 was ascribed to each specimen.

In the case of immunohistochemistry, HAUSP antigen was calculated with a specific rabbit antibody (Cell Signaling Technology; Danvers, MA, USA). The antigen–antibody complexes were then visualized through the avidin-biotin-peroxidase complex kit (Elite kit; Vector Laboratories, Burlingame, ca., USA). The sections were finally counterstained with hematoxylin and mounted. Images of sections were photographed with a digital camera (Nikon, Tokyo, Japan).

### Statistical Analysis

All data were analyzed using Student’s *t*-test with Bonferroni adjustment for multiple comparisons and are presented as mean ± SD. Statistical analyses were conducted using the SPSS (Version 12.0) statistical software program (SPSS, Chicago, IL, USA). Differences were considered significant at p < 0.05. For survival, data were graphed and analyzed by the product limit method of Kaplan and Meier, using the log-rank (Mantele-Cox) test for comparisons using GraphPad Prism (version 5.0, La Jolla, CA, USA).

## Data Availability Statement

The original contributions presented in the study are included in the article/**Supplementary Material**. Further inquiries can be directed to the corresponding authors.

## Ethics Statement

All participants, as both patients and human normal, provided informed consent, and the patients were enrolled based on their diagnosis prior to chemotherapy treatment. The patients/participants provided their written informed consent to participate in this study. All animal-related procedures were reviewed and approved by the Institutional Animal Care and Use Committee of the Hanyang University (protocol 2020-0060).

## Author Contributions

J-SK, HK, EC, S-JM, and SJ performed the molecular experiments and generated and documented the data. JJ and C-SY designed and conceptualized the research, supervised the experimental work, analyzed the data, and wrote the manuscript. All authors contributed to the article and approved the submitted version.

## Funding

This work was supported by a National Research Foundation of Korea grant funded by the Korea government (MSIP) (grant no. 2019R1I1A2A01064237 and 2021R1A4A5032463) and the research fund of Hanyang University (HY-2021).

## Conflict of Interest

The authors declare that the research was conducted in the absence of any commercial or financial relationships that could be construed as a potential conflict of interest.

## Publisher’s Note

All claims expressed in this article are solely those of the authors and do not necessarily represent those of their affiliated organizations, or those of the publisher, the editors and the reviewers. Any product that may be evaluated in this article, or claim that may be made by its manufacturer, is not guaranteed or endorsed by the publisher.
