# Supplementary material for: PE_PGRS38 Interaction With HAUSP Downregulates Antimycobacterial Host Defense via TRAF6
Source: Front Immunol. 2022 Apr 28;13:862628. doi: 10.3389/fimmu.2022.862628 (PMC9095961; doi:10.3389/fimmu.2022.862628)
Supplement: Supplementary file 1 [file DataSheet_1.docx]

**SUPPLEMENTARY Materials and Methods**

***Study population of human normal and patients with asthma and lung cancer*** Two types of samples were included in this study. All participants, as both patients and human normal, provided informed consent, and the patients were enrolled based on their diagnosis prior to chemotherapy treatment. We collected lung biopsy from 20 individuals: 10 human lung (median age 57.4 ± 12.9 y; male 50.5%) and 10 asthma patients (median age 54.7 ± 18.3 y; male 49.5%); 10 human lung (median age 49.6 ± 19.3 y; male 40.8%) and 10 lung cancer patients (median age 53.6 ± 19.6 y; male 47.4%). All participants provided written informed consent regarding the use of their clinical data for research purposes.

***Phagosome Purification***

Phagosomes were formed by the internalization of latex beads in culture medium at 37 °C for the indicated times. Cells were then washed in PBS on ice, disrupted in homogenization buffer (3 mM imidazole, pH 7.4, containing 8.55% [w/w] sucrose, 2 mM phenylmethylsulfonyl fluoride, 1 mg/ml chymostatin, 1 mg/ml E-64, 1 mg/ml leupeptin, 1 mg/ml pepstatin) by nitrogen cavitation for 20 min at 300 psi in a bomb (Parr Instrument) at 4°C. After centrifugation at 1,500 rpm for 7 min to remove nuclei and unbroken cells, the supernatants phagosomal compartments were subjected to stepwise sucrose gradient centrifugation. Centrifugation was carried out at 24,000 rpm for 1 hr in a SW41 Beckman swinging rotor. The phagosomes fractions were collected from the 10%/25% sucrose interface. The collected phagosomes fractions were washed with PBS. The purity of the final phagosomes fractions was evaluated by electron microscopy.

**SUPPLEMENTARY Figures**

**
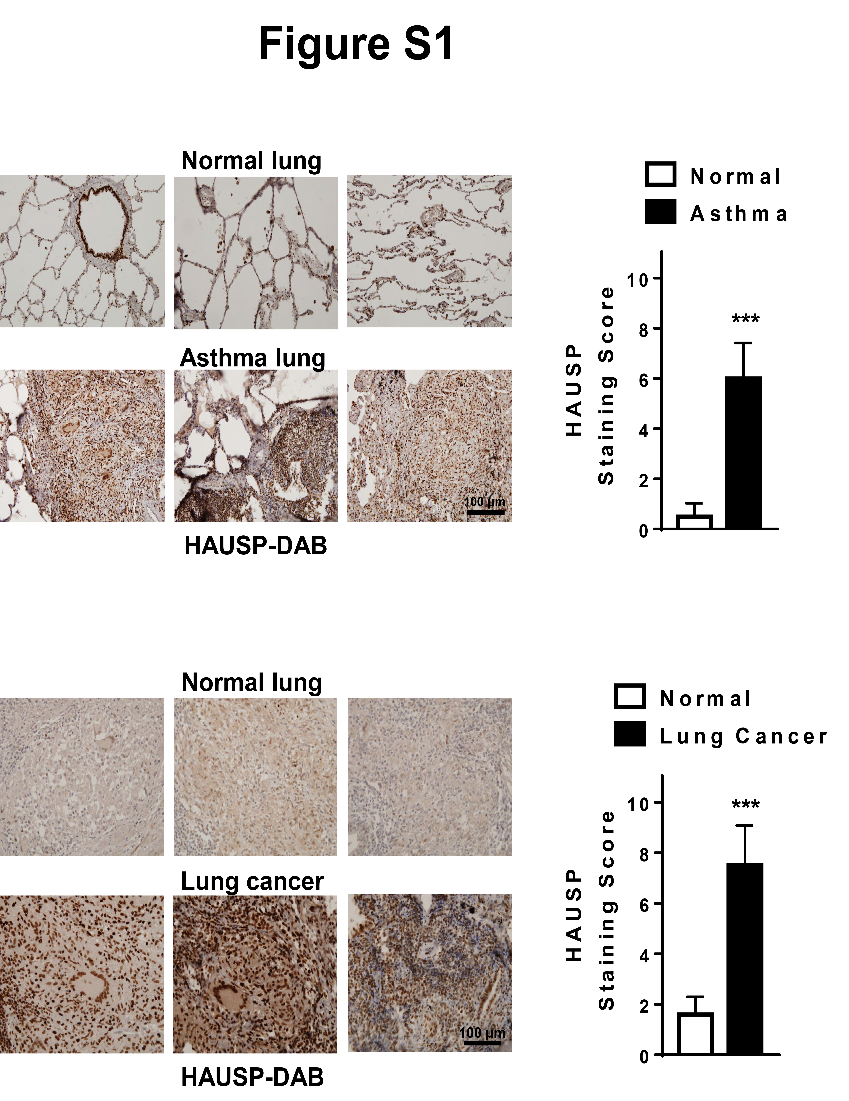
**

**Figure S1. The expression of HAUSP was increased in asthma and lung cancer patients.** (A and B) Immunohistochemistry images of HAUSP in the lungs of normal and asthma or lung cancer patients (Left) and staining score of HAUSP (Right). Scale Bar, 100μm. The data are representative of three independent experiments with similar results (A and B). Significant differences (***p < 0.001) are compared with the lungs of normal group (two-tailed Student’s t-test with Bonferroni adjustment).


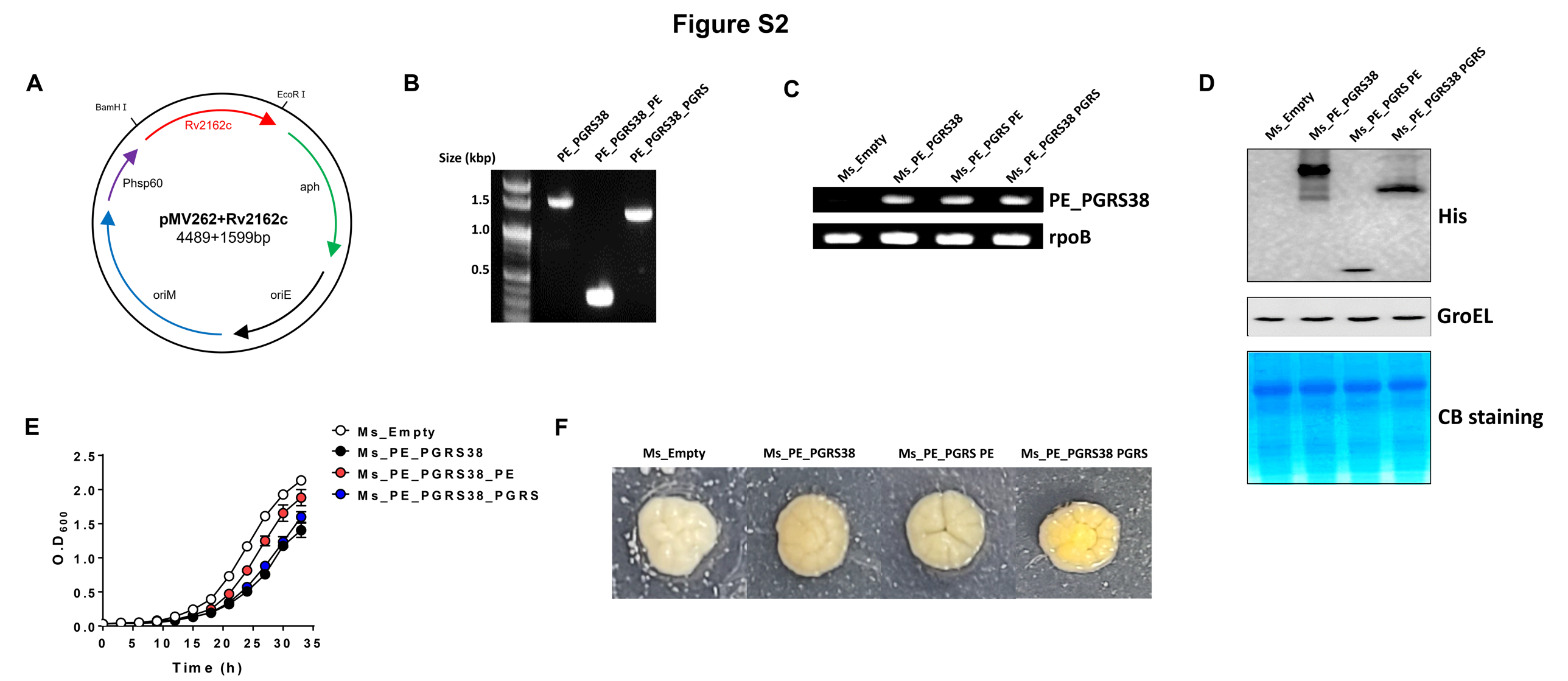


**Figure S2. Construction of recombinant *M. smegmatis*.** (A) Schematics of pMV262 with PE_PGRS38 plasmid. (B) Amplicon of PE_PGRS38, PE_PGRS38_PE, and PE_PGRS38_PGRS. (C) Identification of mRNA expression of PE_PGRS38, PE_PGRS38_PE, PE_PGRS38_PGRS in *M. smegmatis*. rpoB is used as normalization. (D) Identification of protein expression of PE_PGRS38, PE_PGRS38_PE, PE_PGRS38_PGRS in *M. smegmatis*. WCLs were used for IB with αGroEL and Coomassie blue staining is used as normalization. (E) Growth curve of Ms_Empty, Ms_PE_PGRS38, Ms_PE_PGR38_PE, and Ms_PE_PGRS38_PGRS. The OD_600_ were measured at an interval of 3h (F) The morphology of Ms_Empty, Ms_PE_PGRS38, Ms_PE_PGRS38_PE, and Ms_PE_PGRS38_PGRS in 7H10 agar supplemented with kanamycin (50 μg/mL).


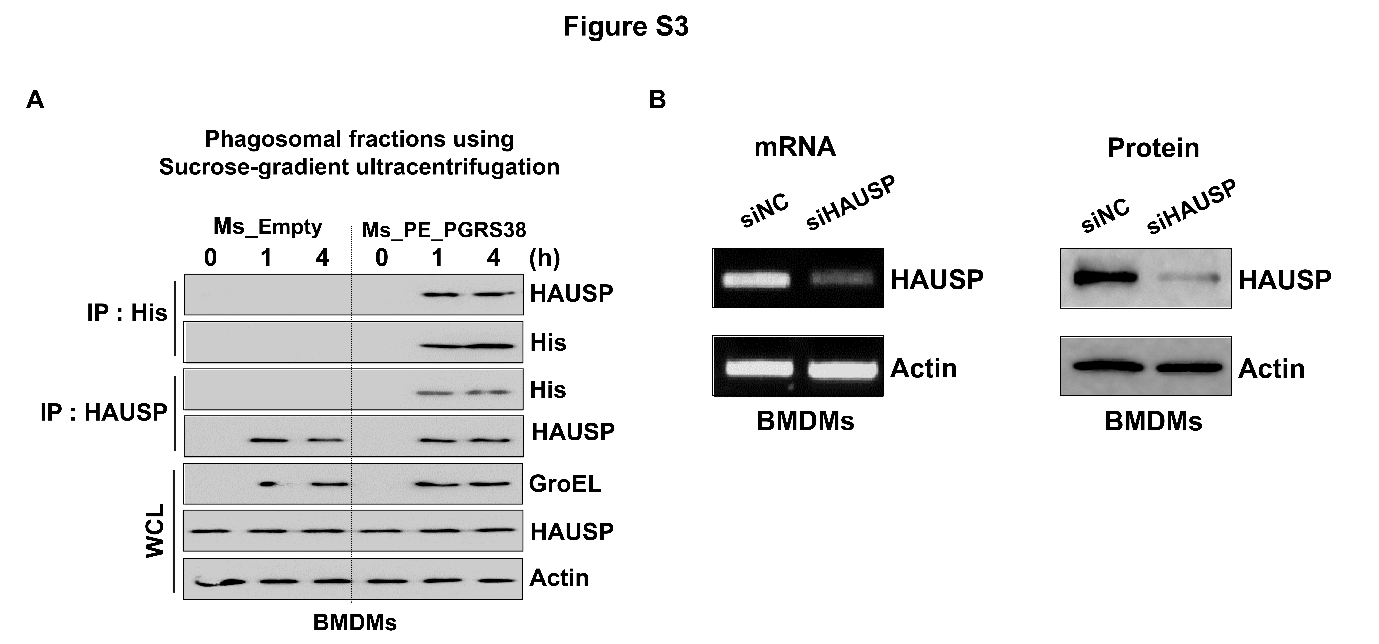


**Figure S3. Phagosomal fractions and validation of HAUSP knockdown by siHAUSP in BMDMs.** (A) BMDMs were infected with Ms_Empty or Ms_PE_PGRS38 for indicated times. BMDMs were fractionated for phagosome by using sucrose-gradient ultracentrifugation and used for IP with αHis or αHAUSP, followed by IB with αHis and αHAUSP. WCLs were used for IB with αGroEL, αHAUSP and α Actin. (B) BMDMs were treated with siNC or siHAUSP (20μm) for 2 days. After 2 days, BMDMs were used for validation of level of HAUSP mRNA and protein. Actin is used as normalization.

**
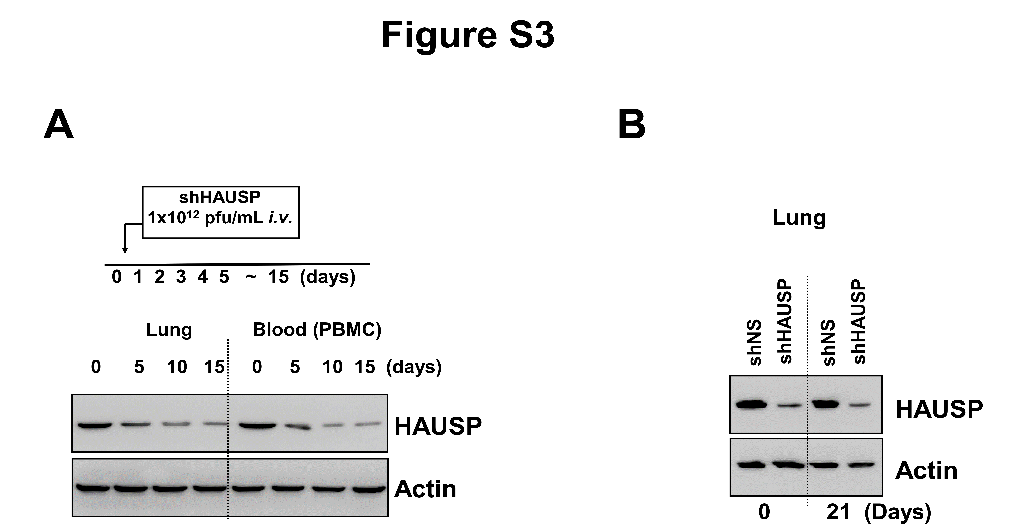
**

**Figure S4. Validation of HAUPS knockdown by shHAUSP in mice.** (A and B) Mice were intravenously infected by lenti-shNS or lenti-shHAUSP (1x10^12^ pfu/ml) for indicated time. Lung or PBMC of mice were used for IB with αHAUSP and αActin.
